# Blood‐derived product therapies for SARS‐CoV‐2 infection and long COVID

**DOI:** 10.1002/mco2.426

**Published:** 2023-11-15

**Authors:** Junzheng Wu, Huichuan Yang, Ding Yu, Xiaoming Yang

**Affiliations:** ^1^ Chengdu Rongsheng Pharmaceuticals Co., Ltd. Chengdu China; ^2^ China National Biotec Group Company Ltd Beijing China; ^3^ Beijing Tiantan Biological Products Co., Ltd. Beijing China

**Keywords:** anti‐inflammatory, blood‐derived products, immunomodulatory, long COVID, passive immunotherapy, SARS‐CoV‐2 infection

## Abstract

Severe acute respiratory syndrome coronavirus 2 (SARS‐CoV‐2) is capable of large‐scale transmission and has caused the coronavirus disease 2019 (COVID‐19) pandemic. Patients with COVID‐19 may experience persistent long‐term health issues, known as long COVID. Both acute SARS‐CoV‐2 infection and long COVID have resulted in persistent negative impacts on global public health. The effective application and development of blood‐derived products are important strategies to combat the serious damage caused by COVID‐19. Since the emergence of COVID‐19, various blood‐derived products that target or do not target SARS‐CoV‐2 have been investigated for therapeutic applications. SARS‐CoV‐2‐targeting blood‐derived products, including COVID‐19 convalescent plasma, COVID‐19 hyperimmune globulin, and recombinant anti‐SARS‐CoV‐2 neutralizing immunoglobulin G, are virus‐targeting and can provide immediate control of viral infection in the short term. Non‐SARS‐CoV‐2‐targeting blood‐derived products, including intravenous immunoglobulin and human serum albumin exhibit anti‐inflammatory, immunomodulatory, antioxidant, and anticoagulatory properties. Rational use of these products can be beneficial to patients with SARS‐CoV‐2 infection or long COVID. With evidence accumulated since the pandemic began, we here summarize the progress of blood‐derived product therapies for COVID‐19, discuss the effective methods and scenarios regarding these therapies, and provide guidance and suggestions for clinical treatment.

## Graphical abstract

The development and application of blood‐derived product therapeutics are essential in mitigating the considerable damage caused by SARS‐CoV‐2 infection and long COVID. Various blood‐derived products, including COVID‐19 convalescent plasma, COVID‐19 hyperimmune globulin, recombinant anti‐SARS‐CoV‐2 neutralizing immunoglobulin G, intravenous immunoglobulin (IVIG), and albumin, are investigated for their potential use in therapeutic applications. Furthermore, the insights acquired from the administration of blood‐derived products during the ongoing COVID‐19 pandemic can serve as a reference for treating other potentially acute infectious diseases.

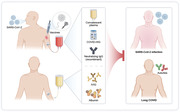

## 1 INTRODUCTION

Since the initial onset of severe acute respiratory syndrome coronavirus 2 (SARS‐CoV‐2) infections, data collected by the World Health Organization show that prototype and mutant SARS‐CoV‐2 strains have infected approximately 800 million people and caused approximately 7 million deaths worldwide,^1^ and these numbers could be serious underestimations. However, SARS‐CoV‐2 will not stop evolving; circulating variants that have attracted attention, such as Omicron subvariants BA.2.86 and EG.5,^2^ and highly mutated viral strains that evade the protective effects of vaccines and exhibit higher infectivity than the original strain or earlier variants, continue to emerge.^3^ Furthermore, increased transmissibility does not necessarily imply a reduction in the virulence of SARS‐CoV‐2 and may lead to increased disease severity or even mortality.^4^, ^5^ Although the virulence of the Omicron strain, which is currently prevalent worldwide, has decreased, its high transmissibility and ability to evade the immune system have caused large‐scale outbreaks of infection.^6^ Considering the high infection rate of coronavirus disease 2019 (COVID‐19), the number of severely‐to‐critically ill patients within the total population of infected patients should not be overlooked.

An observational cohort clinical study conducted in the Netherlands revealed that more than 12% of patients with COVID‐19 may be affected by long‐term health issues (long COVID),^7^ and the incidence of long COVID is positively correlated with the severity of the acute phase of COVID‐19.^8^ Importantly, long COVID can affect several organ systems, causing adverse outcomes and persistent disease for several months to years.^8^, ^9^ The considerable cumulative burden on health caused by COVID‐19 also remains a concern, and long COVID results in 80–643 disability‐adjusted life years per 1000 recovered individuals.^10^ The care needs of individuals with long COVID may persist in the long‐term; however, postinfectious recovery or treatment guidelines for patients with COVID‐19 are unavailable.

More transmissible SARS‐CoV‐2 variants may emerge and continue to be transmitted on a large scale in the population, with the risk of sequelae remaining elevated. Therefore, methods to control SARS‐CoV‐2 infection and treat long COVID should be a high priority. One effective approach is the use of blood‐derived products to treat patients with acute SARS‐CoV‐2 infection or long COVID.^11^, ^12^, ^13^ Blood‐derived products refer to therapeutic substances derived from human blood donations or plasma, including whole blood and other blood components for transfusion, and plasma‐derived medicinal products.^14^ Although the clinical safety of these blood‐derived products has been confirmed, their therapeutic effectiveness against COVID‐19 has shown variability in clinical studies.^15^, ^16^, ^17^, ^18^

Attempts to develop COVID‐19 drugs and therapies have been actively pursued^19^, ^20^, ^21^; however, reviews of blood‐derived product therapies remain lacking. Therefore, considering the immune pathophysiology during different stages of COVID‐19, we discuss the mechanisms of different blood‐derived products as treatments, focusing on their application progress as virus‐targeting passive immunotherapies and immune modulators. Furthermore, we summarize the clinical findings of different blood‐derived product therapies and possible factors influencing their efficacy, and provide guidance for their clinical application against SARS‐CoV‐2 infection and long COVID.

## 2 DEVELOPMENT OF BLOOD‐DERIVED COMPONENTS DURING THE COVID‐19 PANDEMIC

Whole blood primarily comprises plasma, erythrocytes, leukocytes, and platelets, which can be separated into different components, that is, blood‐derived products, and used to address child mortality and maternal health, improve the life expectancy and quality of life of patients with life‐threatening inherited disorders, and provide support for complex medical and surgical procedures.^14^, ^22^ Although transfusion therapy is a commonly used treatment method, this intervention remains associated with several potential risks. Hence, providing patients with appropriate blood components (such as packed red blood cells, platelet concentrate, and plasma‐derived proteins) to replace whole blood is the direction pursued by modern blood transfusion therapy.^23^, ^24^

The plasma composition is complex and includes massive amounts of water, various electrolytes, and thousands of proteins.^25^ Plasma‐derived protein components, including intravenous immunoglobulin (IVIG), albumin, and clotting proteins, have been prepared as medicinal products and are used in the treatment of chronic and acute life‐threatening diseases such as hemorrhagic, autoimmune, and hereditary angioedema.^26^ Plasma fractionation technology is mature, but the manufacturing of plasma‐derived medicinal products remains limited by insufficient global supply of plasma.^27^

**FIGURE 1:**
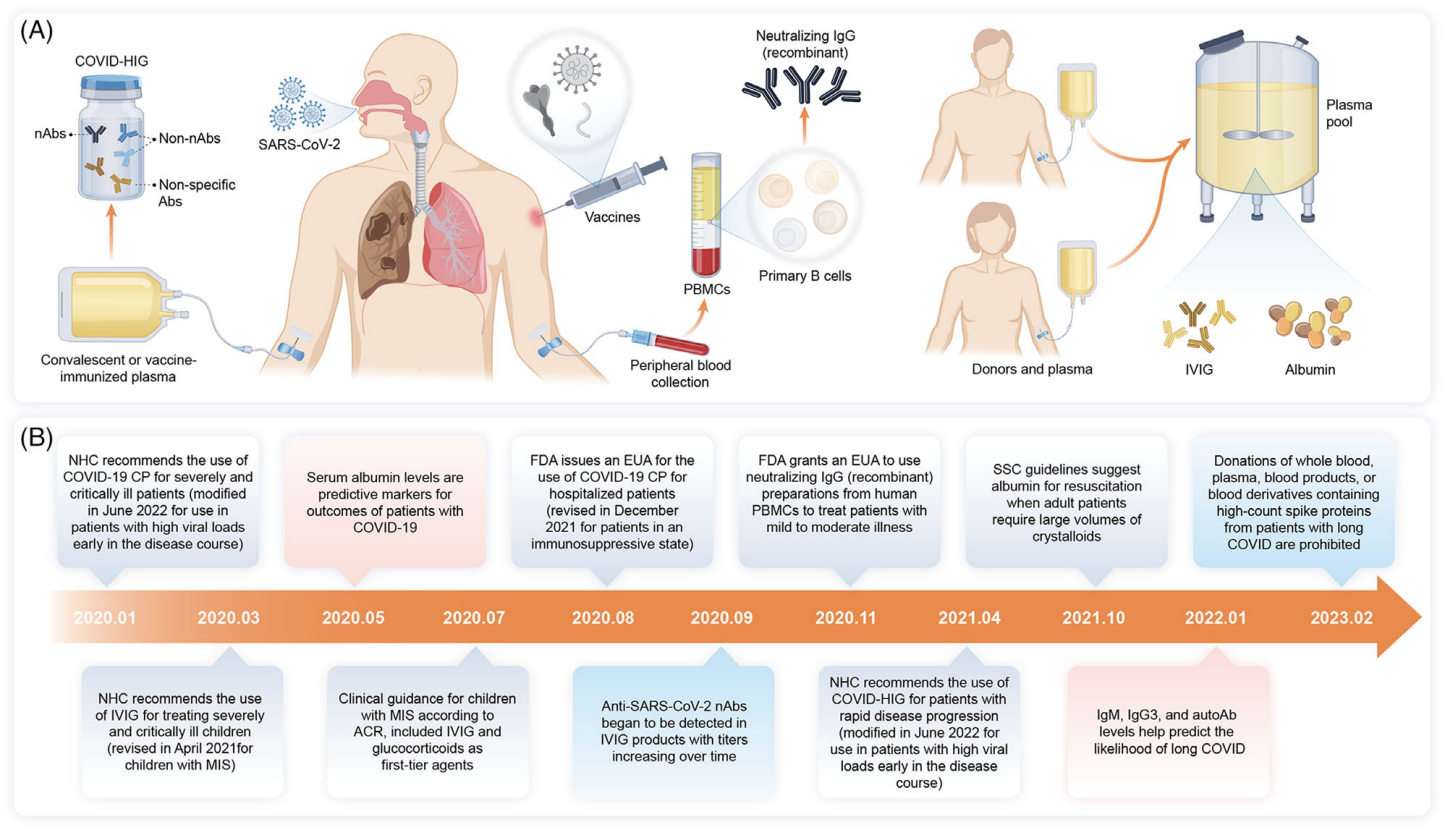
Application of blood‐derived components in the context of the ongoing coronavirus disease 2019 (COVID‐19) pandemic. (A) SARS‐CoV‐2‐targeting and non‐SARS‐CoV‐2‐targeting blood‐derived products. (B) Historical timeline of using blood‐derived components for prognosis prediction and clinical treatment of patients with COVID‐19. COVID‐HIG, COVID‐19 hyperimmune globulin; nAbs, neutralizing antibodies; non‐nAbs, non‐neutralizing antibodies that do not impede SARS‐CoV‐2 infectivity but bind to SARS‐CoV‐2; nonspecific Abs, nonspecific antibodies that cannot bind to SARS‐COV‐2; autoAbs, autoantibodies; PBMCs, peripheral blood mononuclear cells; IVIG, intravenous immunoglobulin; CP, convalescent plasma; IgG, immunoglobulin G; IgM, immunoglobulin M; NHC, National Health Commission of the People's Republic of China; MIS‐C, multisystem inflammatory syndrome in children; ACR, American College of Rheumatology; U.S. FDA, United States Food and Drug Administration; EUA, emergency use authorization; SSC, Surviving Sepsis Campaign.

Following viral infection or vaccination, the host rapidly develops immune responses against the antigen, wherein antibodies and cell‐mediated immunity alter the protein composition of the blood.^28^ Therefore, blood‐derived products can be categorized based on whether the donors have been previously exposed to SARS‐CoV‐2‐related antigens, namely SARS‐CoV‐2‐targeting and non‐SARS‐CoV‐2‐targeting products. SARS‐CoV‐2‐targeting blood‐derived products include COVID‐19 convalescent plasma (CP), COVID‐19 hyperimmune globulin (COVID‐HIG), and recombinant anti‐SARS‐CoV‐2 neutralizing immunoglobulin G (IgG). Non‐SARS‐CoV‐2‐targeting blood‐derived products include IVIG and human serum albumin (Figure 1A).

### 2.1 Advances in SARS‐CoV‐2‐targeting blood‐derived products

The COVID‐19 pandemic required rapid evaluation and development of blood‐derived products at an unprecedented speed (Figure 1B). COVID‐19 CP neutralizes the virus and is beneficial; furthermore, CP may be the fastest treatment strategy for acute infectious diseases that also ensures the safety of patients.^29^ Since SARS‐CoV‐2 was found to be highly contagious, China has focused on the safety and efficacy of COVID‐19 CP.^30^, ^31^ In January 2020, the National Health Commission of the People's Republic of China (NHC) published the *Diagnosis and Treatment Protocol for COVID‐19 (Trial Version 4)*, recommending COVID‐19 CP for severely or critically ill patients with COVID‐19.^30^ The Emergency Use Authorization (EUA) of COVID‐19 CP was issued by the U.S. Food and Drug Administration (U.S. FDA) in August 2020 for hospitalized patients with COVID‐19.^32^ In December 2021, the U.S. FDA revised these guidelines, suggesting the use of COVID‐19 CP for patients with immunosuppressive diseases or those receiving immunosuppressive treatment, with a preference for CP with high neutralizing antibody (nAb) titers.^12^, ^32^ In June 2022, the NHC also recommended using COVID‐19 CP for the antiviral treatment of patients with rapid disease progression, high viral loads, and high‐risk factors during the early course of COVID‐19.^33^ Two other SARS‐CoV‐2‐targeting blood‐derived products were approved for treatment following COVID‐19 CP approval, as the drug registration processes or production required more time than those of CP.

In April 2021, the NHC recommended the use of COVID‐HIG to treat patients with mild or severe COVID‐19 showing rapid disease progression.^34^ COVID‐HIG can be prepared from COVID‐19 CP, COVID‐19 vaccine immune plasma (VP), and COVID‐19 hyperimmune animal plasma (serum).^35^, ^36^, ^37^ COVID‐HIG is prepared primarily using commercial IVIG production methods,^35^, ^36^ and the NHC exclusively recommends the use of COVID‐HIG derived from human immune plasma.^34^ Anti‐SARS‐CoV‐2 neutralizing IgG (recombinant) can also be derived from the peripheral blood mononuclear cells (PBMCs) of convalescent patients or SARS‐CoV‐2‐vaccinated donors,^38^, ^39^ and the U.S. FDA first issued the EUA for neutralizing IgG (recombinant) derived from PBMCs of convalescent patients with COVID‐19 in November 2020.^12^

Both COVID‐HIG and neutralizing IgG (recombinant) can be derived from blood products after vaccination, and may have broad and potent anti‐SARS‐CoV‐2 efficacy.^38^ From this perspective, the protection provided by vaccines is not only limited to the prevention of SARS‐CoV‐2 infection, but also aids in the treatment of COVID‐19. COVID‐19 vaccines that have received marketing approval or been approved for EUA can be classified into mRNA, inactivated COVID‐19, nonreplicating adenovirus vector, and recombinant S protein subunit vaccines.^40^ Fully approved COVID‐19 vaccines were designed to protect against the original SARS‐CoV‐2 strain; however, they cannot provide effective protection against Omicron and its variants. Therefore, to reduce the losses caused by the global spread of Omicron variants, updated mRNA vaccines including a monovalent component (XBB.1.5) were granted EUA by the U.S. FDA.^41^ In China, the trivalent recombinant S protein subunit vaccine protects against both the Delta and Omicron (XBB.1.5 + BA.5 + Delta) variants and was approved for EUA.^42^

Of particular concern is the likelihood that more virulent or contagious SARS‐CoV‐2 variants will emerge, and the U.S. FDA anticipates that COVID‐19 vaccine ingredients may require to be updated annually, similar to those of seasonal influenza vaccines.^41^ Thus, more promising vaccines against SARS‐CoV‐2 variants have been explored to provide a greater level of protection.^43^, ^44^

### 2.2 Advances in non‐SARS‐CoV‐2‐targeting blood‐derived products

Since March 2020, the NHC has recommended the use of IVIG to treat severely and critically ill children.^45^ In July 2020, the American College of Rheumatology (ACR) recommended IVIG and glucocorticoids as first‐tier agents for pediatric COVID‐19 in their clinical guidance for treating multisystem inflammatory syndrome in children (MIS‐C).^11^ MIS‐C is a rare condition associated with SARS‐CoV‐2 that usually occurs 2−6 weeks post‐infection and can cause inflammation of different internal and external body parts, which can be serious or even fatal.^46^ In April 2021, the NHC revised the guidelines, recommending that IVIG be used for MIS‐C treatment.^34^ Regarding another non‐SARS‐CoV‐2‐targeting blood‐derived product, the Surviving Sepsis Campaign (SSC) suggested the use of albumin combined with large volumes of crystalloids over the use of standalone crystalloids for adult patients with septic shock or sepsis.^47^ Although septic shock may also occur in critically ill patients with COVID‐19, considering the cost and limited availability of albumin, the SSC does not recommend routine treatment with albumin during the acute resuscitation of adult patients with COVID‐19.^48^, ^49^

### 2.3 Blood‐derived components of concern due to SARS‐CoV‐2 emergence

The levels of certain blood proteins in patients with COVID‐19 can predict clinical outcomes and the emergence of long COVID (Figure 1B). Serum albumin levels are related to clinical outcomes in patients with COVID‐19, with lower serum albumin levels indicating a higher risk of mortality. Since May 2020, serum albumin levels have been suggested as indicators of disease severity and prognosis for patients with COVID‐19.^50^, ^51^, ^52^ Similarly, low levels of total IgG3 and IgM antibodies in blood samples from patients have been found to predict the risk of long COVID independently.^53^ Furthermore, elevated levels of autoantibodies (autoAbs) may reflect a subclinical situation and suggest the risk of autoimmune diseases in patients with long COVID.^54^

Since September 2020, commercially available IVIG has shown only a weak neutralization effect against SARS‐CoV‐2; however, the titers of nAbs have been increasing over time.^55^, ^56^ Considering that IVIG has been used for the treatment of both SARS‐CoV‐2 infection and secondary disease after infection (long COVID), this increase in titers should be considered beneficial for patients with COVID‐19. However, the circulating spike (S) protein of SARS‐CoV‐2 may persist in the plasma of patients with long COVID.^57^ Consequently, donations of whole blood, plasma, blood products, or blood derivatives containing high levels of S proteins are prohibited by law in the U.S. state of Montana.^58^

## 3 RATIONALE FOR USING BLOOD‐DERIVED PRODUCTS IN COVID‐19 TREATMENT

The onset of the COVID‐19 pandemic significantly impacted blood donation due to illness and public health restrictions, but the demand for blood‐derived products remains high.^59^, ^60^ Currently, except for a few plasma‐derived proteins, such as factors VIII and IX and von Willebrand factor, that can be industrially produced through gene recombinant expression, Igs and albumin can still only be obtained via separation from donated plasma.^61^, ^62^ Therefore, the development of blood‐derived products that either target or do not target SARS‐CoV‐2 can potentially alleviate the current situation of inappropriate use and shortage of blood products.

Blood‐derived products have been used for the treatment of infectious diseases, with CP being administered in the late 19th century to limit the spread of viral diseases, including polio, measles, and influenza.^63^ Subsequently, fresh‐frozen plasma has been explored to aid in the rehabilitation of patients with septic shock^64^; supplemental plasma coagulation factors have also been used to ameliorate the prognosis of patients with Ebola hemorrhagic fever.^65^ At the early stages of the COVID‐19 outbreak, expectations were high for blood‐derived products.^66^, ^67^ Based on the long history of blood‐derived products and discoveries over the past 3 years, in this section, we summarized the potential mechanisms of blood‐derived products for COVID‐19 to provide a rationale for refining this therapeutic strategy.

### 3.1 Protective and pathological functions of Fc‐dependent SARS‐CoV‐2 antibodies effector functions in SARS‐CoV‐2 infection

After infection with SARS‐CoV‐2, the host mounts antibody‐mediated responses to eliminate the virus. The B cells of the germinal centers are stimulated by the antigens of SARS‐CoV‐2 to differentiate into mature plasma cells, secreting virus‐specific IgG, IgM, and IgA antibodies to control the infection. Several structural SARS‐CoV‐2 proteins, including the S, membrane, and nucleocapsid (N) proteins, elicit a strong humoral immune response by the host.^68^ Among them, the receptor‐binding domain (RBD), N‐terminal domain, and stem helix and fusion peptide of the S protein facilitate viral infection^69^; therefore, the S‐specific antibodies produced by the host are considered nAbs.

Host cell‐secreted IgG, IgM, or IgA antibodies are primarily directed against the S and N proteins of SARS‐CoV‐2, and extensive research is underway to identify these antibodies against specific epitopes on these proteins.^70^, ^71^, ^72^ The median time for seroconversion for SARS‐CoV‐2‐specific IgA antibodies is approximately 1 week after symptom onset,^73^, ^74^ slightly earlier than that for IgG and IgM antibodies (approximately 2 weeks after symptom onset).^68^, ^75^ However, the duration of virus‐specific IgM and IgA antibodies is shorter than that of IgG antibodies, and the levels of the IgA and IgM antibodies decrease more rapidly than those of IgG antibodies.^76^ Furthermore, the persistence of IgG antibodies targeting different viral epitopes differs. Our previous study revealed that even 24 months after recovery, approximately 75% of recovered patients continued to exhibit detectable IgG antibodies against RBD (RBD‐IgG), whereas only approximately 25% exhibited IgG antibodies against the N protein (NP‐IgG).^71^ This may be because a longer duration of nAb responses helps protect the host against reinfection.

**FIGURE 2:**
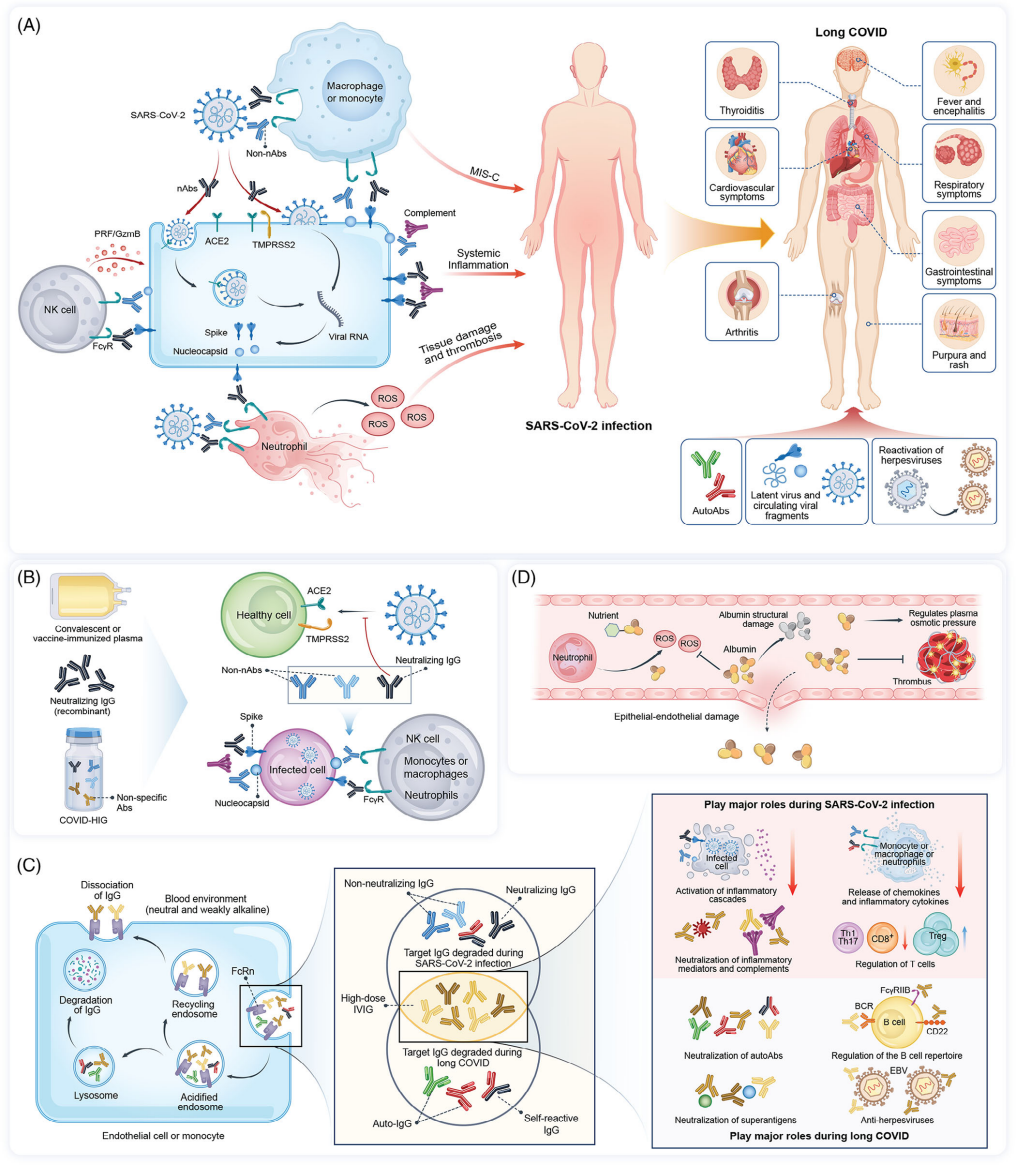
Blood‐derived products targeting pathophysiological factors in COVID‐19. (A) Dysregulated SARS‐CoV‐2 specificity and/or autoantigen antibodies, reservoirs of SARS‐CoV‐2, and herpesvirus reactivation contribute to the pathogenesis of exacerbated disease in patients with SARS‐CoV‐2 infection or long COVID. (B) SARS‐CoV‐2‐targeting blood‐derived products block viral infection of cells or simultaneously recognize and eliminate infected cells via Fc‐mediated effector functions. (C) Metabolic regulation of SARS‐CoV‐2 specificity and autoantigen antibodies by IVIG and anti‐inflammatory and immunomodulatory properties of IVIG among patients with SARS‐CoV‐2 infection or long COVID. (D) Albumin exhibits antioxidant properties against antioxidative stress and maintains plasma osmotic pressure and an anti‐hypercoagulation state. COVID‐19, coronavirus disease 2019; SARS‐CoV‐2, severe acute respiratory syndrome coronavirus 2; nAbs, neutralizing antibodies; non‐nAbs, non‐neutralizing antibodies that do not impede the infectivity of SARS‐CoV‐2 but are capable of binding to SARS‐CoV‐2; ACE2, angiotensin‐converting enzyme 2; TMPRSS2, transmembrane serine protease 2; PRF, perforin; GzmB, granzyme B; FcRn, neonatal Fc receptor; FcγR, Fc receptor for IgG; ROS, reactive oxygen species; COVID‐HIG, COVID‐19 hyperimmune globulin; IVIG, intravenous immunoglobulin; Auto‐IgG, autoimmune IgG; Treg, regulatory T cell; BCR, B cell receptor; FcγRIIB, Fcγ receptor IIB; CD22, cluster of differentiation‐22; EBV, Epstein–Barr virus.

SARS‐CoV‐2 may be blocked by nAbs prior to the invasion of healthy host cells. However, when nAbs fail to block viral entry, the S and N proteins on the surface of infected host cells could be recognized by antibodies binding to SARS‐CoV‐2. Although these antibodies fail to block virus‐infected cells, they can exert antiviral effects via Fc‐mediated effector functions^77^ (Figure 2A, left). Both nAbs and non‐nAbs bind to SARS‐CoV‐2 to form an immune complex, which is bound by complement proteins or recognized by Fc receptors (FcRs) on the surface of host immune cells.^78^ Subsequently, the virus is cleared by complement‐dependent or cell‐mediated cytotoxic activity (Figure 2A, left).

IgG antibodies are the predominant antibody isotype in human serum.^79^ Human FcRs for IgG (FcγRs) comprise activating and inhibitory receptors that transmit signals via intracellular immunoreceptor tyrosine‐based activation and inhibitory motifs, respectively.^80^ The humoral immune responses of patients with COVID‐19 play key roles in suppressing SARS‐CoV‐2 infection. However, critically ill patients often exhibit high nAb titers, wheras most recovered patients produce relatively low levels of nAbs,^81^ raising speculation regarding the pathogenic consequences of antibody responses.^70^, ^82^

Following infection with SARS‐CoV‐2, antibodies mediate immune functions via Fc‐dependent effectors, showing both protective and pathogenic outcomes, and the reasons for these contradictory results are complex. The Fc‐mediated effector functions of antibodies after infection may contribute to increased inflammation levels and pathological damage, ultimately exacerbating the disease.^83^ Children with MIS display persistent and increased levels of SARS‐CoV‐2‐specific IgG antibodies, and high inflammatory monocyte‐activation activity.^84^ Nonspecific activation of B cells occurs in MIS‐C, resulting in increased autoantigen responses that trigger self‐attack.^85^ Furthermore, SARS‐CoV‐2 uses the property of antibodies binding to FcγR to invade macrophages or monocytes; although infected macrophages and monocytes undergo pyroptosis to terminate the infection, they also secrete interleukin‐1β and interleukin‐18, triggering systemic inflammation^86^ (Figure 2A, left). In patients with high disease severity, SARS‐CoV‐2‐specific antibodies contribute to extensive complement deposition in multiple organs, associated with high systemic inflammation and pathological damage^87^ (Figure 2A, left). Elevated neutrophil activation increases the release of neutrophil extracellular traps (NETs), referred to as NETosis, and reactive oxygen species (ROS), with NETs persisting longer in critically ill patients.^88^ However, adverse clinical outcomes after respiratory viral infection are also commonly associated with high levels of NETs.^89^, ^90^ NETs cause endothelial injury and thrombosis, whereas ROS can structurally damage albumin, impairing its ability to bind nutrient ligands and leading to the dysfunction of the red blood cells and lungs^78^, ^91^, ^92^ (Figure 2A, left).

### 3.2 Possible mechanisms underlying long COVID

Long COVID has a series of different symptoms, and the corresponding mechanisms may also be diverse, including the long‐term adverse effects of autoAbs. Patients with COVID‐19 exhibit enhanced antibody responses against self‐antigens, indicated by autoAbs against immunomodulatory proteins (type I interferons, cytokines, and chemokines), members of the exoproteome (extracellular and secreted proteins), and phospholipids.^93^, ^94^ Moreover, these autoAbs persist after recovery during long COVID.^95^ Some SARS‐CoV‐2‐specific antibodies bind to the virus and cross‐react with self‐antigens.^96^ This phenomenon possibly intensifies the disease risk of patients with COVID‐19. Viral infection induces various autoimmune diseases by initiating immunodeficiency in patients with COVID‐19, thereby disrupting their ability to maintain self‐tolerance and causing the immune system to recognize self‐antigens. In long COVID, the autoAbs may lead to conditions such as MIS‐C, systemic lupus erythematosus, immune thrombocytopenia, cardiovascular disorders (an elevated heart rate, myocarditis, vasculitis, and thrombosis), neurological symptoms (small‐fiber polyneuropathy and autoimmune encephalitis), gastrointestinal symptoms (abdominal pain and loss of appetite), cutaneous involvement (maculopapular eruptions, urticarial lesions, and chilblains), autoimmune thyroid diseases, and arthritis^97^, ^98^, ^99^ (Figure 2A, right).

Furthermore, the persistent reservoirs of SARS‐CoV‐2 in tissues (circulating SARS‐CoV‐2 RNA fragments and SARS‐CoV‐2 superantigens) and herpesvirus reactivation are factors potentially involved in the development of long COVID^8^, ^54^, ^100^ (Figure 2A, right). Clinical monitoring for SARS‐CoV‐2 is usually carried out in the respiratory or gastrointestinal tract, and the virus is no longer detectable after a short period.^101^ However, the virus replicates in multiple organ systems, and SARS‐CoV‐2 RNA fragments and antigens can persist for long period in some patients.^102^ This prolonged existence may explain why long COVID symptoms affect different organ systems, including the cardiovascular, nervous, respiratory, and digestive tracts.^103^ Herpes viruses are more likely to be reactivated following SARS‐CoV‐2 infection, and the reactivation of Epstein–Barr virus (EBV) is correlated with the progression of long COVID.^54^

### 3.3 Mechanisms of blood‐derived products for SARS‐CoV‐2 infection and long COVID

All SARS‐CoV‐2‐targeting blood‐derived products contain nAbs that block the binding of the virus to angiotensin‐converting enzyme 2 (ACE2), whereas COVID‐19 CP and COVID‐HIG also contain polyclonal non‐nAbs against the NP, membrane, and envelope proteins or other viral epitopes, exhibiting Fc‐mediated inhibitory effects on infected cells^104^ (Figure 2B). Non‐SARS‐CoV‐2‐targeting blood‐derived products possess anti‐inflammatory, immunomodulatory, antioxidant, and anticoagulatory properties,^105^, ^106^, ^107^ making them potential candidates to alleviate an excessive inflammatory response in patients with COVID‐19 and treat patients with autoAb‐induced long COVID. The Fc of IgG antibodies is crucial for prolonging the half‐life of IVIG, as IgG antibodies bound to neonatal Fc receptors (FcRns) in endosomes are not degraded by lysosomes, and eventually transported out of endothelial cells to re‐enter the circulation.^108^ After the FcRns are saturated with IVIG, SARS‐CoV‐2‐specific antibodies causing excessive inflammation in severely and critically ill patients and autoAbs that persist after recovery during long COVID are both rapidly metabolized because they are unable to bind sufficient FcRns^109^ (Figure 2C, left). Excessive inflammatory responses caused by the Fc‐mediated effects of SARS‐CoV‐2‐specific antibodies are alleviated after IVIG administration.

Both F(ab')2 and Fc endow IVIG with excellent anti‐inflammatory and immunomodulatory properties, producing diverse mechanisms through which IVIG treatment alleviates COVID‐19. For example, IVIG lowers the levels of inflammatory factors, blocks complement proteins to reduce the inflammatory response,^110^ and induces specific antibodies that mediate granulocyte death.^111^ IVIG also regulates the T cell pool by inducing the apoptosis of Th1 and Th17 cells and increases the expansion of regulatory T cells while simultaneously reducing the number of CD8^+^ T cells and inhibiting the activation of antigen‐presenting cells^112^ (Figure 2C, right). Generally, high doses of IVIG are used clinically to treat autoimmune diseases; the total dosage every 4 weeks may reach 1−2 g/kg body weight.^113^, ^114^ IVIG also neutralizes certain autoAbs,^115^ cytokines, complements,^116^ and viral superantigens^117^; reduces B cell activation and proliferation; and promotes the apoptosis of B cells, including those producing autoAbs^118^ (Figure 2C, right).

IVIG contains antibodies that cross‐react with EBV, and its clinical use in patients with EBV reactivation is beneficial.^119^ IVIG produced before the COVID‐19 pandemic has been reported to cross‐react with SARS‐CoV‐2 antigens, although IVIG did not exhibit neutralizing activity.^120^ However, with the increasing number of patients recovered from COVID‐19 or the vaccination of healthy plasma donors, neutralizing activity has been detected since then in a wide range of IVIG, and nAb titers have been increasing over time.^55^, ^56^, ^121^ Additionally, IVIG is derived from mixed plasma obtained from thousands of individuals, it possesses broad antibody profiles and the potential to simultaneously respond to long COVID induced by latent SARS‐CoV‐2, circulating viral fragments, autoAbs, and EBV reactivation (Figure 2C, right). The diverse structures and functions of antibodies endow IVIG with the characteristics of multiple mechanisms, IVIG can effectively intercept various pathogenic factors and enhance protection against secondary infection. Therefore, the clinical value of IVIG therapy in the field of infectious and autoimmune diseases has gained increased attention during the COVID‐19 pandemic. When the dose of COVID‐HIG is similar to that of high‐dose IVIG, COVID‐HIG should provide the protective effects derived from SARS‐CoV‐2‐targeting antibodies and exhibit the effects of IVIG (Figures 2B and C).

Hypoalbuminemia has also been observed in patients with COVID‐19 and is much more pronounced in severe and critical cases. This condition may be derived from epithelial–endothelial damage resulting from the SARS‐CoV‐2 infection, triggering the onset of pulmonary capillary leak syndrome.^52^ Human serum albumin acts as an antioxidant and the primary carrier of various endogenous and exogenous ligands; thus, it is the primary agent that maintains the plasma redox state and osmotic pressure.^106^ The increased numbers of ROS‐positive neutrophils induced by SARS‐CoV‐2 infection cause oxidative stress in the blood, structurally damaging the albumin. Higher ROS‐positive neutrophil numbers are related to fatal outcomes in severe and critically ill patients with COVID‐19.^92^ Therefore, supplementation with albumin may alleviate excessive oxidative stress in critically ill patients. Furthermore, albumin exhibits anticoagulant properties and is a therapeutic option for suppressing serum hypercoagulability in patients with COVID‐19.^49^, ^107^

## 4 CLINICAL APPLICATION OF SARS‐COV‐2‐TARGETING BLOOD‐DERIVED PRODUCTS AGAINST COVID‐19

Passive immunotherapy has been a safe form of treatment to control infectious diseases for over a century.^122^ COVID‐19 CP, COVID‐HIG, and neutralizing IgG (recombinant) all passively enhance host immunity to SARS‐CoV‐2. Therapy with COVID‐19 CP or COVID‐HIG is generally considered safe and has demonstrated potential in COVID‐19 treatments.^16^, ^123^

### 4.1 SARS‐CoV‐2‐targeting blood‐derived products in COVID‐19

Treatment with COVID‐19 CP, COVID‐HIG, or neutralizing IgG (recombinant) provides passively transferred SARS‐CoV‐2 antibodies to susceptible recipients. Due to the high number of patients with COVID‐19, COVID‐19 CP is easily obtained and one of the earliest therapeutic strategies available.^124^, ^125^ Additionally, the cost of COVID‐19 CP is lower than that of COVID‐HIG and neutralizing IgG (recombinant), hence it is used globally.^126^ More than 100 clinical trials have been initiated to evaluate the effectiveness of COVID‐19 CP for the prevention and treatment of COVID‐19. Clinical setting scenarios include, but are not limited to, exploring patients with different disease severity, different disease progression stages when starting dosing, different age groups of patients, patients with different immune status or functions, inpatients or outpatients, and whether combined antiviral drugs are used.^127^ However, after more than 3 years of research, the safety of COVID‐19 CP has been generally recognized, yet consensus regarding its effectiveness has not been achieved.

COVID‐HIG is prepared from COVID‐19 hyperimmune plasma; several clinical studies on COVID‐HIG have been published.^16^, ^37^, ^128^, ^129^ The clinically used dose range of COVID‐19 CP‐derived COVID‐HIG is 0.15−0.4 g/kg; the dose of COVID‐HIG derived from COVID‐19 hyperimmune animal plasma is lower, 4 mg/kg (two doses). The common denominator is that the use of COVID‐HIG is safe and no concerning antibody‐dependent enhancement effects have been observed.^130^ Among the VP‐derived COVID‐HIGs, only the VP obtained after inactivated vaccination has been used to produce COVID‐HIG; however, the results of clinical studies have not been published.^36^ In theory, the efficacy and safety of VP‐derived COVID‐HIG could be well predicted based on the efficacy of the vaccine against SARS‐CoV‐2.

In COVID‐19 CP, the IgG antibody levels far exceed those of IgM or IgA antibodies and play a decisive role in the therapeutic effect.^131^ Furthermore, COVID‐HIG primarily consists of IgG with only trace amounts of IgM or IgA.^35^, ^36^ Hence, the antibody type of recombinant, commercial nAbs, or those being investigated for treating COVID‐19 should be IgG.^12^, ^72^ Various neutralizing IgG (recombinant) targeting S protein demonstrated effectiveness in preventing and treating COVID‐19, and have therefore been granted EUA by the U.S. FDA.^132^, ^133^

### 4.2 Factors influencing the effectiveness of SARS‐CoV‐2‐targeting blood‐derived products

**FIGURE 3:**
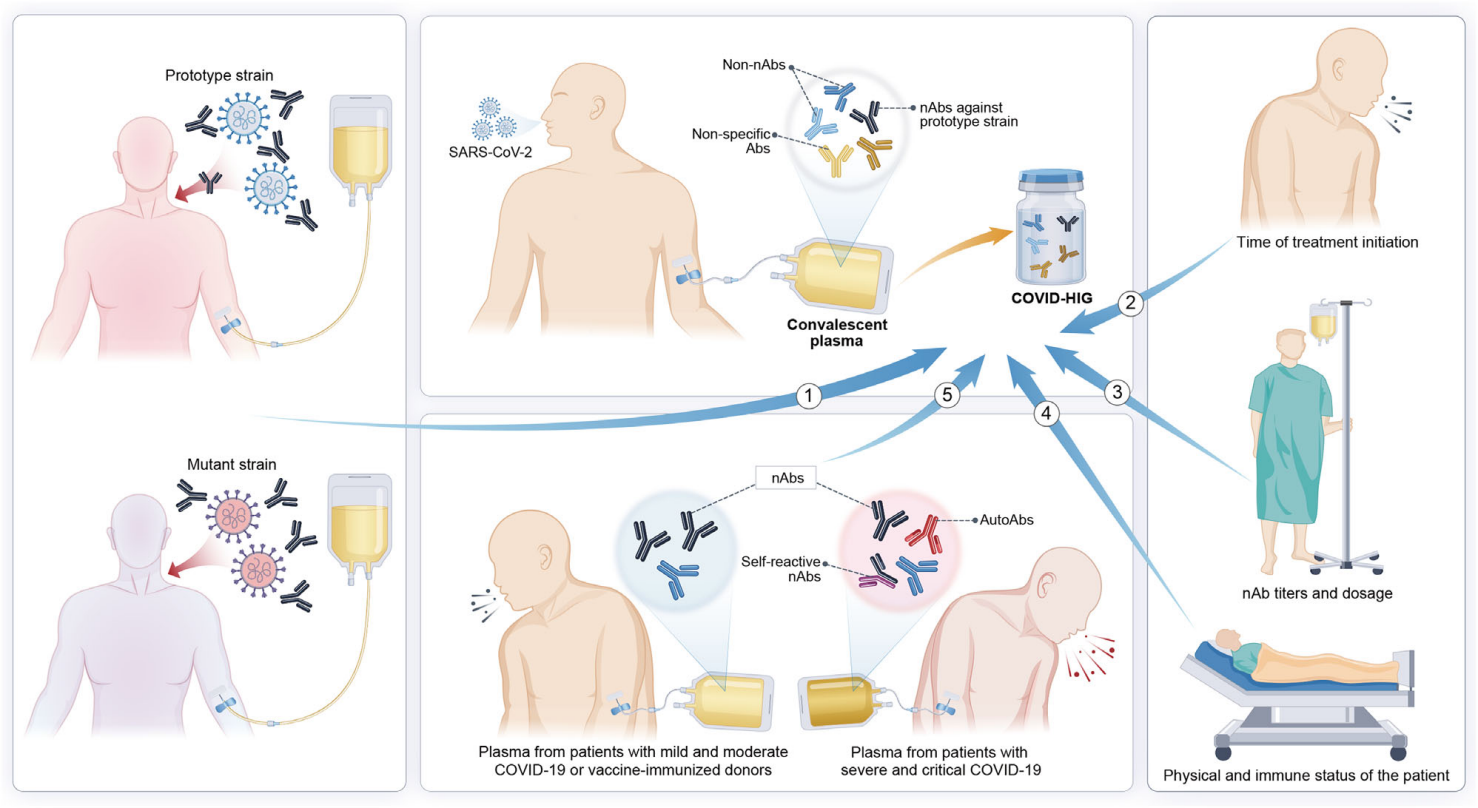
Factors influencing the effectiveness of COVID‐19 CP or COVID‐HIG. Therapeutic effects of CP obtained from individuals who have recovered from COVID‐19 varied depending on the prototype or mutant strain of infection. The timing of treatment initiation, physical condition and immune status of patients, nAb titers of CP or COVID‐HIG, and diversity in antibody profiles of CP are important factors that affect clinical efficacy. COVID‐19, coronavirus disease 2019; CP, convalescent plasma; COVID‐HIG, COVID‐19 hyperimmune globulin; SARS‐CoV‐2, severe acute respiratory syndrome coronavirus 2; nAbs, neutralizing antibodies; non‐nAbs, non‐neutralizing antibodies that do not impede the infectivity of SARS‐CoV‐2 but are capable of binding to SARS‐CoV‐2; autoAbs, autoantibodies.

The clinical trial results of SARS‐CoV‐2‐targeting blood‐derived products have not always been consistently positive^16^, ^17^, ^134^; to better understand the potential factors that may influence the effectiveness of SARS‐CoV‐2‐targeting blood‐derived products, we analyzed data from large clinical studies (Table 1). Several key factors can influence treatment efficacy, including the timing of treatment initiation, nAb titers of COVID‐19 CP or COVID‐HIG, and the physical and immune status of patients receiving these treatments. Additionally, the effect of SARS‐CoV‐2 mutants that evade host immunity on COVID‐19 CP or COVID‐HIG efficacy may be particularly significant (Figure 3).

**TABLE 1:** Comparison of information reported from COVID‐19 CP and COVID‐HIG clinical trials.

Product types/period	Participants	Circulating strains	Administration start time	Results	Trial No./References
COVID‐19 CP					
June 4–October 25, 2020	Older adults (65 years or older) with mild COVID‐19 (*n* = 160)	Prototype^a^	39.6 h after symptom onset	The progression of COVID‐19 was reduced^b^	NCT04479163/^131^
May 28–August 27, 2020	Hospitalized adults with severe COVID‐19 (*n* = 334)	Prototype	8 d after symptom onset	There were no significant improvements in the clinical status or overall mortality of patients	NCT04383535/^134^
April 4–July 4, 2020	Hospitalized adult patients with COVID‐19 (*n* = 3082)	Prototype^c^	10 d after diagnosis (with mechanical ventilation)	There was no significant effect on the risk of death^d^	NCT04338360/^135^
			5.4 d after diagnosis (without mechanical ventilation)	High antibody levels in CP reduced the risk of death^d^	
April 21–October 27, 2020	Hospitalized adult patients with severe COVID‐19 (*n* = 223)	Prototype	11 d after symptom onset	There was a significant reduction in 28‐d mortality	NCT04359810/^136^
May 28, 2020–January 15, 2021	Hospitalized patients with COVID‐19 (*n* = 11,558)	Prototype and Alpha^e^	10.5 d after symptom onset (first dose)	Survival or other prespecified clinical outcomes did not improve^f^	NCT04381936/^15^
November 10, 2020–July 28, 2021	Adult outpatients with mild COVID‐19 (*n* = 376)	Prototype and Alpha^g^	4.4 d after symptom onset	CP did not ameliorate COVID‐19 progression or reduce viral load	NCT04621123/^137^
June 3, 2020–October 1, 2021	Adult outpatients with recent‐onset COVID‐19 (*n* = 1225)^h^	Prototype, Alpha, and Delta^i^	6 d after symptom onset	CP reduced the risk of COVID‐19 progression resulting in hospitalization	NCT04373460/^17^
COVID‐HIG					
Human CP HIG/October 8, 2020–February 10, 2021	Hospitalized adults with COVID‐19 without end‐organ failure (*n* = 593)	Prototype, Alpha, Eta, and Lambda^j^	8 d after symptom onset	hIVIG had no clinical benefits for hospitalized patients^k^	NCT04546581/^16^
Human CP HIG/June 19, 2020–February 3, 2021	Severely or critically ill patients with COVID‐19 (*n* = 50)	Prototype and Alpha^l^	8 d after symptom onset	C‐IVIG increased survival and reduced the risk of disease progression^m^	NCT04521309/^129^
Human CP HIG/April–July 2021	Severely immunocompromised hospitalized adults with COVID‐19 (*n* = 18)	Prototype and Alpha	9 d after symptom onset	COVIG may reduce the risk of severe COVID‐19	NL9436 (trialsearch.who.int)/^128^
Equine serum HIG/August 1–October 26, 2020	Hospitalized adults with moderate to severe COVID‐19 (*n* = 245)	Prototype	6 d after symptom onset	Good safety profile but primary endpoint not met	NCT04494984/^37^

Abbreviations: C‐IVIG, hyperimmune anti‐COVID‐19 intravenous immunoglobulin; COVID‐19, coronavirus disease 2019; COVIG, antisevere acute respiratory syndrome coronavirus 2 (SARS‐CoV‐2) hyperimmune globulin; CP, convalescent plasma; d, days.; HIG, hyperimmune intravenous immunoglobulin; hIVIG, hyperimmune intravenous immunoglobulin to SARS‐CoV‐2.

^a^ SARS‐CoV‐2 genomic surveillance began in Argentina in May 2020, and SARS‐CoV‐2 variants began to be detected in epidemiologic week 10 of 2021.^138^

^b^ CP infusions have an IgG dose‐dependent effect and plasma with high IgG titers reduces the risk of severe respiratory disease, indicating that IgG is the active therapeutic ingredient in CP.

^c^ From April to July 2020, the U.S. Centers for Disease Control and Prevention reported no prevalent mutant strains.^139^ ^.^

^d^ Patients who received CP transfusion within 3 days of diagnosis exhibited a lower risk of death than those who received CP at 4 or more days after diagnosis.

^e^ The Alpha variant was detected in the UK in September 2020 and became the dominant variant by January 2021.^140^ CP was obtained from blood services in England, Northern Ireland, Scotland, and Wales. Antigenic shifts in SARS‐CoV‐2 strains owing to regional differences may have affected the accuracy of evaluating CP efficacy.

^f^ A Bayesian re‐analysis confirmed that the subgroup of individuals who had not developed endogenous anti‐SARS‐CoV‐2 antibodies benefited from CP treatment.^141^

^g^ The CP samples used in the trial were obtained during the circulation of SARS‐CoV‐2 variants, including B.1, B.1.1, and B.1.177, but the Alpha strain dominated during the second period.

^h^ Approximately 20% of participants were vaccinated against COVID‐19.

^i^ The CP samples used in the trial were mainly collected from donors infected with the prototype SARS‐CoV‐2 strain in 2020.

^j^ In the summer of 2020, the CP samples used to prepare hIVIG were collected in Europe and North America, during which the globally circulating strain was the original strain.^142^ Between October 2020 and February 2021, different mutant strains spread widely in the UK (Alpha),^140^ Israel (Alpha),^143^ and Nigeria (Alpha, Eta)^144^ and rapidly in Europe (non‐UK; Alpha),^145^ Argentina (Lambda),^146^ and USA (Alpha).^139^ ^.^

^k^ Participants without endogenous neutralizing antibodies receiving hIVIG showed lower rates of hospitalization or death only on day 7 compared with the placebo group. However, participants with endogenous neutralizing antibodies exhibited the opposite trend.

^l^ On December 20, 2020, two cases of infection due to the Alpha variant were reported in Pakistan. However, as of February 2021, the Alpha strain accounted only for a small proportion of cases.^147^ ^.^

^m^ Significantly more optimized recovery in terms of survival, hospital stay, and reduction in disease severity was observed in severely ill patients compared to critically ill ones.

#### 4.2.1 Impact of the SARS‐CoV‐2 strain, treatment timing, and patient status on effectiveness

Most sources of COVID‐19 CP or COVID‐HIG currently used in clinical research are obtained based on the SARS‐CoV‐2 prototype strain (Table 1). As listed in Table 1, in cases with no interference from SARS‐CoV‐2 mutants, patients with COVID‐19 who do not progress to the severe inflammatory stage benefit more from treatment with high nAb titers of COVID‐19 CP or COVID‐HIG. During the severe inflammatory response stage in the lungs, the penetration of the antibody into the infected lungs is low^148^; thus, exogenously supplemented antibodies may not be able to effectively penetrate the lung tissue, making it challenging to eliminate the virus.^16^, ^149^ Importantly, COVID‐19 CP is only effective in patients who have not yet progressed to mechanical ventilation,^135^ whereas COVID‐HIG is more beneficial for patients who are severely ill than for those who are critically ill.^129^ For hyperinflammation, large doses of COVID‐HIG may also be beneficial after saturating FcRns, as the primary mechanism of action involves anti‐inflammatory effects rather than viral clearance (Figure 2).

These passive immunotherapies may benefit immunocompromised patients more than those that are immunocompetent^128^, ^150^; this perspective is also shared by the U.S. FDA.^12^ Interestingly, a large clinical trial revealed that among those participants who received COVID‐HIG, those lacking endogenous nAbs exhibited lower rates of hospitalization or death; conversely, participants with endogenous nAbs exhibited the opposite results.^16^ This phenomenon was also reported in COVID‐19 patients with endogenous nAbs undergoing treatment with SARS‐CoV‐2 neutralizing IgG.^148^ Of note, CP therapy is heterogeneous because of the variations in immune responses and disease severity among recovered individuals and their vaccination status against COVID‐19 or infections with different SARS‐CoV‐2 strains.^134^

Neutralizing IgG (recombinant) targeting SARS‐CoV‐2 epitopes have been identified.^72^ However, these SARS‐CoV‐2 epitopes are also highly variable, and the virus may evade antibody‐mediated immune responses through continuous mutations. Thus, the ability of IgG (recombinant) drugs to maintain their neutralizing effect on SARS‐CoV‐2 is a critical evaluation criterion for retaining their EUA by the U.S. FDA.^12^

#### 4.2.2 Impact of the antibody profiles of COVID‐19 CP and COVID‐HIG on treatment effectiveness

Clinical studies have reported opposite conclusions on the therapeutic effects of COVID‐19 CP and COVID‐HIG, possibly due to the heterogeneity of the antibody profiles. The antibody composition within COVID‐19 CP is highly variable, resulting in significant differences in titers of nAbs, and the types and titers of autoAbs. SARS‐CoV‐2 antigenic changes are region‐ and time‐dependent,^151^ but COVID‐19 CP may have been stored for a period and used in different locations. Therefore, owing to differences in regions and usage time after collection, antigenic shifts in SARS‐CoV‐2 strains may increase the risk of unsatisfactory CP efficacy.^151^

The composition of antibodies in CP should also be recognized because it is beneficial to the analysis of the safety and effectiveness of passive COVID‐19 CP therapy. Critically ill patients produce more autoAbs than mild ones, including certain antibodies targeting self‐tissue antigens, which may be responsible for excessive inflammation or certain autoimmune symptoms in long COVID.^54^, ^96^, ^104^ Furthermore, some autoAbs elicit deleterious effects and suppress interferon‐mediated antiviral immune responses.^152^ Paradoxically, patients with critical COVID‐19 also generally produce high titers of nAbs,^153^ and COVID‐19 CP with high nAb titers is considered beneficial.^12^, ^135^ Additionally, IgG antibodies against the S antigen (S‐IgG) and NP‐IgG both exist in COVID‐19 CP.^154^ NP‐IgG is only a binding antibody to SARS‐CoV‐2 because it cannot block virus‐infected cells. The antiviral effect can only be exerted via the Fc effect. Thus, a higher distribution of S‐IgG as nAbs in CP enhances their effectiveness.

COVID‐HIG is produced from COVID‐19 CP; the quality control requirements during its production include screening the titers of nAbs. However, its antibody profiles are complex, and requirements for the control of autoAbs are lacking.^35^, ^155^ Different from COVID‐19 CP, COVID‐HIG has a higher IgG concentration; hence, the maximum clinical usage can far exceed that of COVID‐19 CP.^36^ However, as COVID‐HIG preparation is time‐consuming, considering the rapid mutation characteristics of SARS‐CoV‐2, the antibody profiles in COVID‐HIG will lose the advantage against the current circulating strain.

## 5 CLINICAL APPLICATION OF NON‐SARS‐CoV‐2‐TARGETING BLOOD‐DERIVED PRODUCTS AGAINST COVID‐19

IVIG and human serum albumin are blood‐derived products that do not specifically target SARS‐CoV‐2 but can potentially treat COVID‐19 or long COVID. IVIG improves IgG deficiencies caused by severe infections and sepsis,^156^ and exhibits anti‐inflammatory and immunomodulatory properties, offering passive protection against various pathogens.^157^ Additionally, albumin exhibits anti‐inflammatory, antioxidant, anticoagulatory, and plasma colloid osmotic pressure maintenance properties (Figures 2C and D). These characteristics render IVIG and albumin theoretically relevant to COVID‐19 treatment and have been widely clinically administered^11^, ^158^ or investigated in clinical trials.^18^, ^50^, ^159^

Following the SARS‐CoV‐2 outbreak, IVIG effectiveness was explored in both SARS‐CoV‐2 infection and long COVID clinical trials (Table 2). Similar to COVID‐19 CP and COVID‐HIG, IVIG has not consistently demonstrated positive outcomes in treating COVID‐19. IVIG does not have virus‐targeting properties; hence, the therapeutic efficacy of IVIG is not weakened by SARS‐CoV‐2 mutants. However, COVID‐19 disease caused by different mutant strains is highly variable, and newly circulating strains may cause milder or more severe disease than previously circulating ones.^170^, ^171^ The therapeutic efficacy of IVIG can be affected by the severity of COVID‐19, and IVIG has higher application value for treating patients with severe or critical illness (Table 2).

**TABLE 2:** Overview of non‐SARS‐CoV‐2‐targeting blood‐derived products and respective clinical trials.

Product types	Participants	Dosage/treatment period	Treatment initiation time	Results	References
IVIG					
	Hospitalized adult patients with severe or critical COVID‐19 (*n* = 58)	20 g/d; 5 d	≤48 or >48 h since ICU admission	Administration of IVIG within 48 h of ICU admission shortened the duration of hospital stay and reduced the 28‐d mortality and use of mechanical ventilation	^160^
	Hospitalized adult patients with critical COVID‐19 (*n* = 325)	0.1−0.5 g/kg/d; 5−15 d	≤7 or >7 d since hospital admission	IVIG administration reduced 28‐d mortality only in critically ill patients. Early administration with high‐dose IVIG reduced both 28‐ and 60‐d mortality in critical patients^a^	^161^
	Hospitalized adult patients with severe COVID‐19 (*n* = 850)	10 g/d; 4−15 d	2.8 d since hospital admission	Treatment with IVIG did not reduce 28‐d mortality in severely ill patients	^162^
	Hospitalized adult patients with severe and critical COVID‐19 (*n* = 535)	1.5 g/kg/d; more than 3 d	5 d since ICU admission	High‐dose IVIG administration improved outcomes in severely or critically ill patients; early administration exhibited a more curative effect	^163^
	Hospitalized adult patients with critical COVID‐19 (*n* = 754)	0.5 g/kg/d	11 d since symptom onset	IVIG treatment did not reduce 28‐d mortality in critically ill patients	^164^
	Patients with critical COVID‐19 who required invasive mechanical ventilation for moderate and severe ARDS (*n* = 146)	0.5 g/kg/d; 4 d	Within the first 96 h of invasive mechanical ventilation^b^	No effects on clinical outcomes on day 28	^159^
IgM‐enriched IVIG	Critically ill adult patients with COVID‐19 requiring respiratory support (*n* = 316)	23.2 g/d; 3−4 d	4 d since ICU admission^c^	Early administration (before mechanical ventilation and/or within the first 14 d after the onset of symptoms) may have beneficial effects without involving safety concerns	^165^
IVIG plus glucocorticoids	Inpatients below 21 years with MIS‐C (*n* = 518)	2 g/kg; 1 or 2 doses	After hospital admission	Initial treatment with IVIG and glucocorticoids reduced the risk of new or persistent cardiovascular dysfunction in MIS‐C	^166^
IVIG plus glucocorticoids	Inpatients below 19 years with MIS‐C (*n* = 111)	2 g/kg; 1 or 2 doses	Unknown	IVIG and methylprednisolone administration was associated with improvement in febrile fever course	^167^
	Patients with long COVID exhibiting respiratory, neurologic, or cardiologic symptoms (*n* = 6)	0.5 g/kg/2 weeks; 3 months	101−547 d since symptom onset	IVIG alleviated long COVID symptoms and yielded significant clinical benefits	^168^
	Patients with long COVID exhibiting peripheral neuropathy (*n* = 17)	1.6−2 g/kg/4 weeks	Unknown	Benefits observed in patients receiving repeated IVIG therapy	^169^
	Patient with autoimmune gastrointestinal motility disorder after SARS‐CoV‐2 infection (*n* = 1)	2 g/kg/month; 4 doses	10 months since symptom onset	Symptoms were significantly alleviated after IVIG treatment	^13^
Albumin					
	Patients with hypoalbuminemia and COVID‐19 in ICU (*n* = 114)	Dose unknown; albumin infusion for 3 d or more consecutively	After ICU admission^d^	Albumin infusion downregulated the levels of COVID‐19‐related biomarkers and reduced the risk of death in critically ill patients with hypoalbuminemia	^18^
	Hospitalized adult patients with COVID‐19 (*n* = 29)	80 g/d (first 3 d) and 40 g/d (4 d after); 7 d for maximum	Unknown	Albumin dampened the hypercoagulable state in hospitalized patients with COVID‐19	^107^

Abbreviations: ARDS, acute respiratory distress syndrome; COVID‐19, coronavirus disease 2019; d, days.; ICU, intensive care unit; IVIG, intravenous immunoglobulin; MIS‐C, multisystem inflammatory syndrome in children;SARS‐CoV‐2, severe acute respiratory syndrome coronavirus 2.

^a^ Early administration refers to treatment initiation within 7 days of hospital admission. High‐dose indicates dosage exceeding 15 g/d, equivalent to 0.2−0.3 g/kg/d.

^b^ 8 days, median time from symptom onset to invasive mechanical ventilation.

^c^ 9 days, median time between symptom onset and ICU admission.

^d^ 23 days, average number of days between symptom onset and ICU admission.

### 5.1 Non‐SARS‐CoV‐2‐targeting blood‐derived products in SARS‐CoV‐2 infection

Clinical trial subgroup analyses based on time to IVIG initiation showed that patients who received IVIG earlier after symptom onset showed benefits,^160^, ^161^, ^163^ whereas those who received IVIG treatment at a later stage did not obtain benefits.^159^, ^164^ Nonetheless, the total dose of IVIG administered was inconsistent among different clinical centers, resulting in a wide range of doses and treatment cycles. The recommended administration dosage is 0.1−1.5 g/kg per day with a treatment cycle of 3−15 days (Table 2). However, the treatment seems effective only when the dosage exceeds 0.2 g/kg per day, as dosages below this threshold were ineffective in different clinical trials.^161^, ^162^ A dosage of 0.2−0.5 g/kg per day requires injection for 5 consecutive days, while 1.5 g/kg per day for 3 days has shown benefits for patients. Furthermore, the proportion of IgM in IVIG affected clinical trial outcomes. IgM‐enriched IVIG (IGAM) is more immunomodulatory than traditional IVIG.^172^ Treatment with IGAM has shown beneficial effects, with no safety concerns, in critically ill patients not yet requiring mechanical ventilation and/or within the first 14 days following symptom onset, when administered for 3 or more days.^165^ However, the significant costs and general scarcity of IGAM hinder its widespread use.

Albumin can improve the function of the respiratory, cardiovascular, and central nervous systems in critically ill patients with hypoalbuminemia.^173^ Patients with COVID‐19 exhibiting low serum albumin levels generally have a poor prognosis^50^, ^51^; therefore, albumin administration may benefit patients with COVID‐19 and hypoalbuminemia.^174^ Clinical research has revealed that continuous albumin supplementation for 3 or more consecutive days inhibits the hypercoagulable state of patients with COVID‐19 and improves the prognosis of patients with COVID‐19 and hypoalbuminemia (Table 2).

### 5.2 Non‐SARS‐CoV‐2‐targeting blood‐derived products in long COVID

During the pandemic, children with MIS developed symptoms similar to those with Kawasaki disease. Kawasaki disease is an acute febrile illness with a contagious etiology that primarily affects children younger than 5 years old, and can be treated with IVIG and aspirin.^175^ A similar syndrome may also occur in adults but at a lower rate.^176^ IVIG is generally effective in treating MIS‐C and is currently the most widely accepted treatment, unanimously recommended by the NHC, ACR, and National Institutes of Health.^11^, ^34^, ^177^ High‐dose IVIG administration is typically used to treat MIS‐C, with a dosage of 1−2 g/kg per day (one or two doses),^11^, ^166^, ^167^ similar to the dose of IVIG administered for the treatment of Kawasaki disease.^178^

The approved indications for the use of IVIG include autoimmune diseases such as chronic inflammatory demyelinating polyneuropathy, immune myasthenia gravis and thrombocytopenic purpura, and Kawasaki syndrome.^179^ Furthermore, SARS‐CoV‐2 infection may be a causative factor for multiple autoimmune diseases that manifest as long COVID.^180^, ^181^ Therefore, clinical research on IVIG administration in patients with long COVID has been initiated to address the current lack of effective therapies for this condition. Subsequent clinical trials have revealed that long‐term high‐dose IVIG administration successfully treats patients with long COVID who also exhibit respiratory, neurological, gastrointestinal, or cardiovascular system diseases.^13^, ^168^, ^169^ Additionally, IVIG administration for endocrine and cutaneous issues may also benefit patients with long COVID.^182^, ^183^

## 6 A GUIDE TO BLOOD‐DERIVED PRODUCT THERAPIES FOR SARS‐CoV‐2 INFECTION AND LONG COVID

**FIGURE 4:**
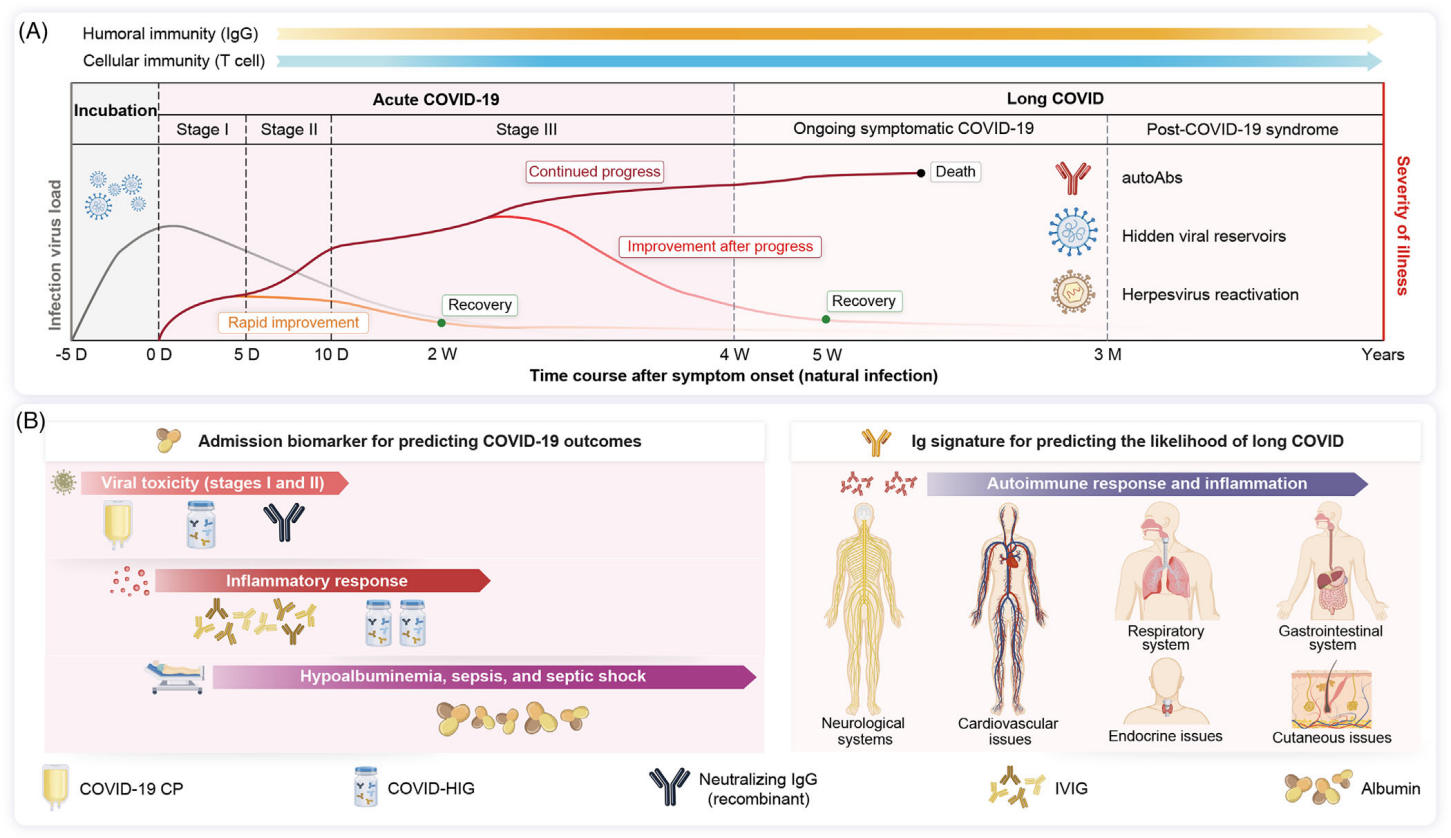
Overview of blood‐derived product therapies for SARS‐CoV‐2 infection and long COVID. (A) Viral load and clinical progression after infection with SARS‐CoV‐2. Symptom onset occurs days after contracting SARS‐CoV‐2. Acute COVID‐19 involves three phases of disease progression, and symptoms lasting more than 4 weeks indicate long COVID, which can be further divided into ongoing symptomatic COVID‐19 and post‐COVID‐19 syndrome. (B) Recommended blood‐derived products for different stages of COVID‐19 progression. COVID‐19 CP, COVID‐HIG, and neutralizing IgG (recombinant) with SARS‐CoV‐2‐targeting properties can be used as antiviral treatments. High‐dose IVIG and COVID‐HIG primarily induce anti‐inflammatory responses. Albumin may ameliorate the prognosis of patients with hypoalbuminemia, sepsis, or septic shock. High‐dose IVIG can be considered for treating long COVID. SARS‐CoV‐2, severe acute respiratory syndrome coronavirus 2; COVID‐19, coronavirus disease 2019; D, day; M, month; autoAbs, autoantibodies; Ig, immunoglobulin; CP, convalescent plasma; COVID‐HIG, COVID‐19 hyperimmune globulin; IVIG, intravenous immunoglobulin.

Considering the complexity of COVID‐19 (including SARS‐CoV‐2 infection and long COVID) and patient heterogeneity, administration of SARS‐CoV‐2‐targeting blood‐derived products (COVID‐19 CP, COVID‐HIG, and neutralizing IgG) and non‐SARS‐CoV‐2‐targeting blood‐derived products (IVIG and albumin) should be carefully timed and dosed to achieve the most efficient COVID‐19 treatment outcomes. To provide improved clinical strategies for blood‐derived product therapies, a clinical phenotype analysis of COVID‐19 was performed according to three disease stages: SARS‐CoV‐2 incubation period, acute COVID‐19, and long COVID (Figure 4A). The incubation period for SARS‐CoV‐2 depends on the viral strain and pre‐existing immunity of the host population, with most individuals exhibiting symptoms approximately 3−5 days post‐infection.^184^ The period of acute COVID‐19 (COVID‑19 symptoms lasting for up to 4 weeks) may be further divided into three levels of increasing severity: stages I (mild), II (moderate), and III (severe). Stage I refers to the early stage of infection, primarily virus proliferation with mild clinical manifestations. Stage II is characterized by pulmonary involvement, wherein virus proliferation is weakened, and local lung inflammation. Stage III represents multi‐stage inflammation throughout the body and manifests as extrapulmonary systemic hyperinflammation syndrome; however, the virus has mostly stopped multiplying at this stage.^184^, ^185^

Long COVID (COVID‑19 conditions continuing for more than 4 weeks) can be subdivided into two stages, namely ongoing symptomatic COVID‐19 and post‐COVID‐19 syndrome.^185^, ^186^ The period of acute symptoms of some patients with COVID‐19 may extend to the ongoing symptomatic COVID‐19 stage, such as inflammation and pathological damage in critically ill patients with COVID‐19 and children with MIS (generally 2−6 weeks after symptom onset).^187^, ^188^

### 6.1 Quality of evidence for blood‐derived product therapies

Based on the evidence‐based medicine approach of the Oxford Center, we assessed the quality of the evidence, and the levels of evidence were rated as 1a, 1b, 1c, 2a, 2b, 2c, 3a, 3b, 4, or 5.^189^ Table 3 lists all blood‐derived products, the type of studies demonstrating effectiveness, and the highest degree of evidence achieved for their effectiveness. The levels of evidence can provide references to formulate clinical recommendations, but low‐level evidence should not be ignored.^200^ Considering the lack of therapeutic strategies for COVID‐19, high‐quality controlled clinical studies remain necessary.

**TABLE 3:** Evidence levels for effectiveness of blood‐derived product therapies.

Product types	Participants	Type of studies	Evidence levels ^a^	Ref.
COVID‐19 CP				
	COVID‐19	Systematic review of RCTs	1a	^190^, ^191^, ^192^
	Immunocompromised patients with COVID‐19	Systematic review of RCTs	1a	^193^
COVID‐HIG				
Human CP HIG	Severely or critically ill patients with COVID‐19	Individual RCT	1b	^129^
	Immunocompromised patients with COVID‐19	Individual RCT	1b	^128^
Human VP HIG	COVID‐19	Physiology, animal research	5	^36^
Neutralizing IgG				
	COVID‐19	Systematic review of RCTs	1a	^194^, ^195^
	COVID‐19 in organ transplant recipients	Systematic review of RCTs	1a	^196^
IVIG				
	Severely and critically ill patients	Systematic review of RCTs	1a	^197^, ^198^
IVIG plus glucocorticoids	MIS‐C	Systematic review of cohort studies	2a	^199^
	Long COVID	Case‐series	4	^168^, ^169^
Albumin				
	Critically ill patients with hypoalbuminemia	Retrospective cohort study	2b	^18^

Abbreviations: COVID‐19, coronavirus disease 2019; COVID‐HIG, COVID‐19 hyperimmune globulin; CP, convalescent plasma; HIG, hyperimmune intravenous immunoglobulin; IVIG, intravenous immunoglobulin; MIS‐C, multisystem inflammatory syndrome in children.; RCT, randomized, controlled trial; VP, vaccine‐immunized plasma.

^a^ The quality of the evidence was rated as level 1a, 1b, 1c, 2a, 2b, 2c, 3a, 3b, 4, or 5 using the evidence‐based medicine approach of the Oxford Center.^189^ ^.^

### 6.2 SARS‐CoV‐2‐targeting blood‐derived product therapies for SARS‐CoV‐2 infection

SARS‐CoV‐2‐targeting blood‐derived products (antiviral therapy) should be administered during periods of rapid viral proliferation, including SARS‐CoV‐2 incubation, stage I, and stage II. Most patients with COVID‐19 produce endogenous anti‐viral antibodies on day 10 post symptom onset,^201^ and patients also enter the aviremic stage at this time.^202^, ^203^ Therefore, early administration of antiviral therapy effectively suppresses viral replication and prevents disease progression (Figure 4B).

The source of COVID‐19 CP is crucial. COVID‐HIG can be derived from COVID‐19 CP, and therefore consideration must be given to the choice of CP as it affects the efficacy of the two aforementioned passive immunotherapies. The high nAb titers of COVID‐19 CP have better treatment efficacy than low titers; thus, obtaining safe COVID‐19 CP with high titers for administration is particularly important. The nAb titers of COVID‐19 CP are affected by several factors, including the time since CP collection from recovered patients, the disease severity of donor patients, and the subtype of SARS‐CoV‐2 strains that the recovered patients were infected with.

The severity of the disease is correlated with nAb titers. Patients with severe and critical infections have higher levels of autoantibodies than those with mild or moderate infections. To ensure safety, CP collected from recovered patients with mild‐to‐moderate disease and high nAb titers are ideal treatment options. A peak in nAb titers is typically observed at 4−5 weeks after the onset of symptoms in recovered patients,^204^ which is the optimal time window for CP collection. Moreover, the antibody profiles of CP collected from recovered patients infected with different mutants may vary; therefore, gathering comprehensive information on recovered patients when collecting CP is essential. COVID‐19 plasma collected close in time and location to patients would be more beneficial.^203^

COVID‐HIG can also be derived from the plasma of healthy donors after COVID‐19 vaccination as it only requires consideration of nAb titers because antibodies produced after receiving the COVID‐19 vaccine are safe.^205^ More importantly, the impact of immune escape by mutant strains is critical for the efficacy of SARS‐CoV‐2‐targeting blood‐derived products, including neutralizing IgG (recombinant) preparations; the immune escape of the globally circulating SARS‐CoV‐2 mutants should continue to be investigated.

Although patient prognosis and recovery should be optimal if viral replication is suppressed during the early stages of infection, in immunocompromised patients, including organ transplant recipients, patients with human immunodeficiency virus infection, and patients with hematologic malignancies, the use of SARS‐CoV‐2‐targeting blood‐derived products should be recommended at all disease progression stages because SARS‐CoV‐2 may be detectable in such patients for 7−8 months or even longer.^206^ The seroconversion rate of immunocompromised patients after receiving the vaccine remains relatively low,^207^ and hence SARS‐CoV‐2‐targeting blood‐derived product therapies can be used as a treatment option that does not rely on the humoral immune response.

### 6.3 Non‐SARS‐CoV‐2‐targeting blood‐derived product therapies for COVID‐19

The onset of stage II (approximately 1 week after symptom onset) is also the median transition time for host cellular and humoral immunity specific to SARS‐CoV‐2.^73^, ^74^, ^82^ At this stage, viral proliferation begins to be specifically and efficiently suppressed; however, inflammation and autoimmunity may intensify (Figure 4A). When the disease progresses to stage II, stage III, and the first half of ongoing symptomatic COVID‐19, viral proliferation further weakens or even stops; however, the influence of inflammation and host hyperimmunity intensifies.^188^ During this time, COVID‐19 treatment should focus on anti‐inflammatory and immune‐regulatory approaches. IVIG or COVID‐HIG can be administered to patients with rapid disease progression, at a dosage of 0.2−0.5 g/kg per day for at least 5 consecutive days. The dose and frequency of administration should be adjusted according to clinical disease severity (Figure 4B).

Theoretically, high‐dose COVID‐HIG should be more effective than IVIG as it eliminates the virus and saturates FcRns (Figure 2). However, the current maximum dose of COVID‐HIG that has been clinically verified does not exceed the maintenance dose of IVIG (0.4−0.6 g/kg; only one dose).^16^, ^128^, ^129^ Considering the economic and practical challenges of obtaining COVID‐HIG, IVIG is recommended for routine administration. Additionally, IVIG at a dose of 1−2 g/kg per day plus glucocorticoids can be administered for 2 consecutive days to treat MIS‐C. Continuous administration of albumin (for 3 consecutive days or more) to adult patients with severely or critically ill COVID‐19, hypoproteinemia, septic shock, or sepsis can restore plasma albumin levels and improve prognosis (Figure 4B).

Only IVIG (among blood‐derived products) is suitable for treating various autoimmune diseases in long COVID. For autoimmune diseases affecting the respiratory, neurological, gastrointestinal, and cardiovascular systems, IVIG can be continuously administered at a dosage of 1−2 g/kg per month for several months or longer to achieve clinical improvement (Figure 4B). For autoimmune diseases associated with other systems or organs, IVIG administration for the aforementioned autoimmune diseases may be referenced for use. However, clinical trials remain necessary to evaluate outcomes.

## 7 CONCLUSIONS

COVID‐19 is a severe and long‐lasting acute infectious disease, the symptoms and causes of which differ during different stages of disease progression (Figure 2A). Blood‐derived products have been widely used to treat COVID‐19 owing to their favorable safety profiles and potential benefits in treating infectious diseases. However, the optimal administration methods and clinical scenarios for using these products in relation to COVID‐19 progression require further investigation (Figure 4A).

SARS‐CoV‐2‐targeting blood‐derived product therapies, such as COVID‐19 CP, COVID‐HIG, and neutralizing IgG (recombinant), are considered passive immunotherapies that enable immediate control of viral infection in the short term, emphasizing the importance of early administration for improved efficacy. After infection, the impact of virus mutations on the immune evasion effects of SARS‐CoV‐2‐targeting blood‐derived products should be a key consideration in their clinical application. Immunosuppressed patients may particularly benefit from the administration of SARS‐CoV‐2‐targeting blood‐derived products. Additionally, for COVID‐19 CP and COVID‐HIG, efforts should be made to obtain COVID‐19 CP with high nAb and low autoAb titers. Contemporaneous COVID‐19 CP with antibodies against current circulating variants exerts a therapeutic effect and should be obtained from individuals with mild‐to‐moderate disease that have recovered. Interestingly, treatment with neutralizing IgG (recombinant) during SARS‐CoV‐2 infection has been associated with a reduced risk of long COVID.^208^ Therefore, this may be an effective strategy for reducing the public health burden caused by long COVID. Whether the other two SARS‐CoV‐2‐targeting blood‐derived products with antiviral effects can reduce the risk of long COVID occurrence remains to be determined.

In the early stages of SARS‐CoV‐2 infection, the innate immune response along with the subsequent sustained adaptive immune response, can suppress the virus. However, if viral replication persists, the SARS‐CoV‐2‐specific IgG antibodies produced by the humoral immune response will recruit additional monocytes/macrophages, neutrophils, and NK cells, thereby triggering further inflammatory responses, ultimately leading to pathological damage. In patients with acute COVID‐19, controlling the immune response to SARS‐CoV‐2 is important for preventing excessive inflammation. Additionally, SARS‐CoV‐2 causes immunodeficiency manifested by the inability of the host immune system to fully distinguish self‐antigens, leading to the development of long COVID, including autoimmune diseases. Thus, the long‐term administration of high‐dose IVIG as an immunomodulator should be considered for correcting host immunodeficiency and benefiting patients.

In addition to being beneficial to the long COVID caused by autoAbs, IVIG may be the only treatment that simultaneously benefits other potential drivers of long COVID, including SARS‐CoV‐2 antigens and RNA fragments and EBV reactivation. Moreover, IVIG is safe and well‐tolerated in long‐term clinical applications.^209^ Non‐SARS‐CoV‐2‐targeting blood‐derived products, including IVIG and human serum albumin, primarily ameliorate host inflammatory responses, regulate the immune system, and alleviate excessive oxidative stress, often requiring large doses to achieve optimal efficacy (Figure 4B). However, neither IVIG nor albumin can prevent the viral spread; thus, non‐SARS‐CoV‐2‐targeting product therapies only have therapeutic effects and should only be considered as supplements to control the COVID‐19 pandemic. Furthermore, high doses of COVID‐HIG may have dual abilities to target SARS‐CoV‐2 and regulate the dysfunctional immune response of the host (Figure 2).

Although various blood‐derived products have been employed for the treatment of acute infectious diseases, their efficacy has not always been consistent during the ongoing COVID‐19 pandemic. Immune evasion following hypermutation of SARS‐CoV‐2, along with the heterogeneity of disease progression and immune status of patients, has presented challenges in determining the therapeutic efficacies of blood‐derived products. Our recommendations are brief theoretical guidelines based on the mechanisms underlying SARS‐CoV‐2 infection and persistent symptoms, combined with the results of clinical trials, to assist patients who may benefit from matched blood‐derived product therapies. Moreover, the experience and lessons acquired from administering blood‐derived product therapies to treat COVID‐19 can serve as a valuable reference for the development of therapies for other potentially emerging acute infectious diseases.

## AUTHOR CONTRIBUTIONS

X. M. Y., J. Z. W., and D. Y. conceptualized the review. J. Z. W., Y. D., and H. C. Y. drafted the manuscript. All authors approved the final submitted version of the manuscript.

## CONFLICT OF INTEREST STATEMENT

J. Z. W. and D. Y. are paid employees of Chengdu Rongsheng Pharmaceuticals Co., Ltd. D. Y. is an employee of Beijing Tiantan Biological Products Co., Ltd. H. C. Y. and X. M. Y. are paid employees of China National Biotec Group Company Ltd (CNBG). These companies are involved in the development of COVID‐HIG; CNBG is involved in the development of anti‐SARS‐CoV‐2 neutralizing IgG (recombinant). The views presented here should not be considered endorsements of any specific products or company.

## ETHICS STATEMENT

Not applicable.

## Data Availability

Not applicable.
